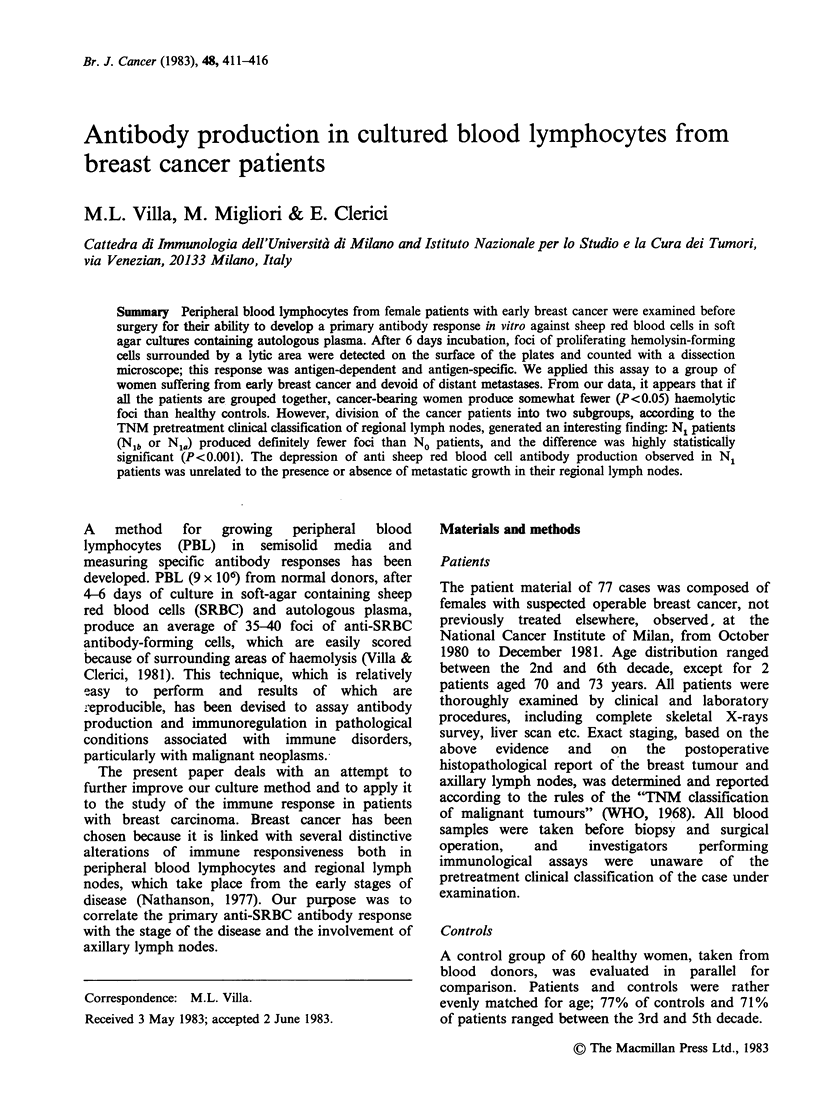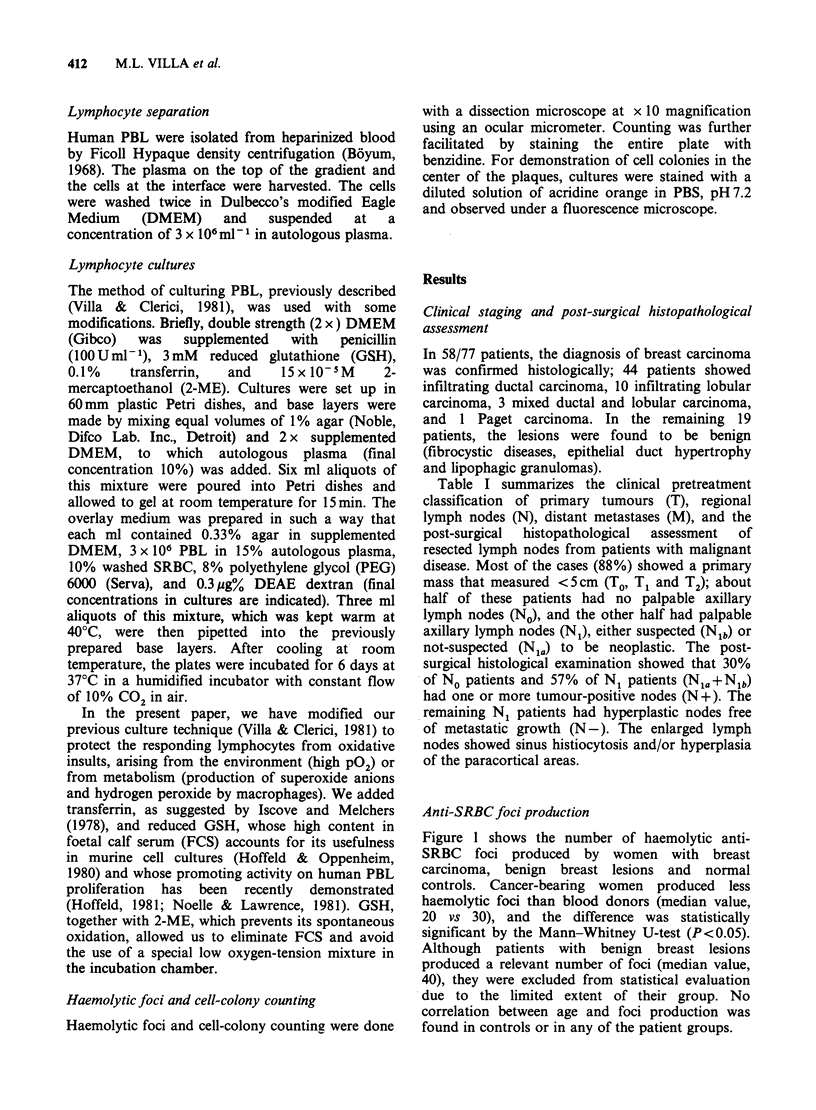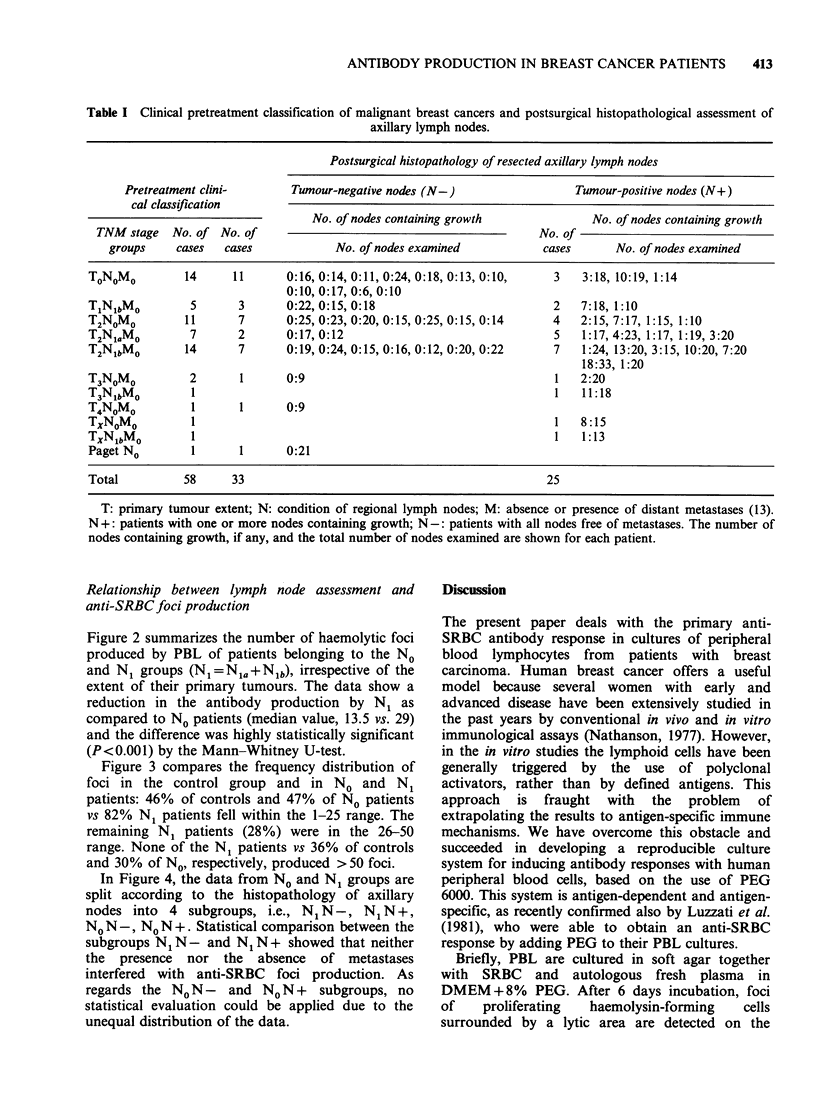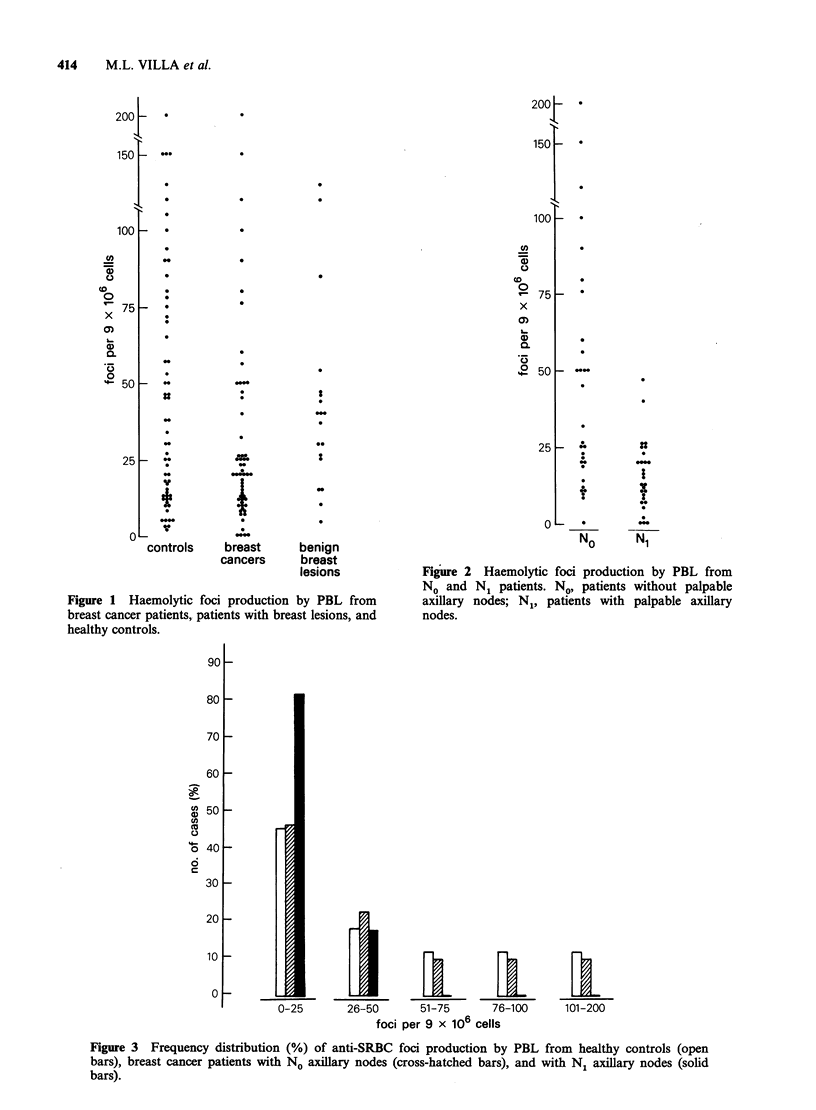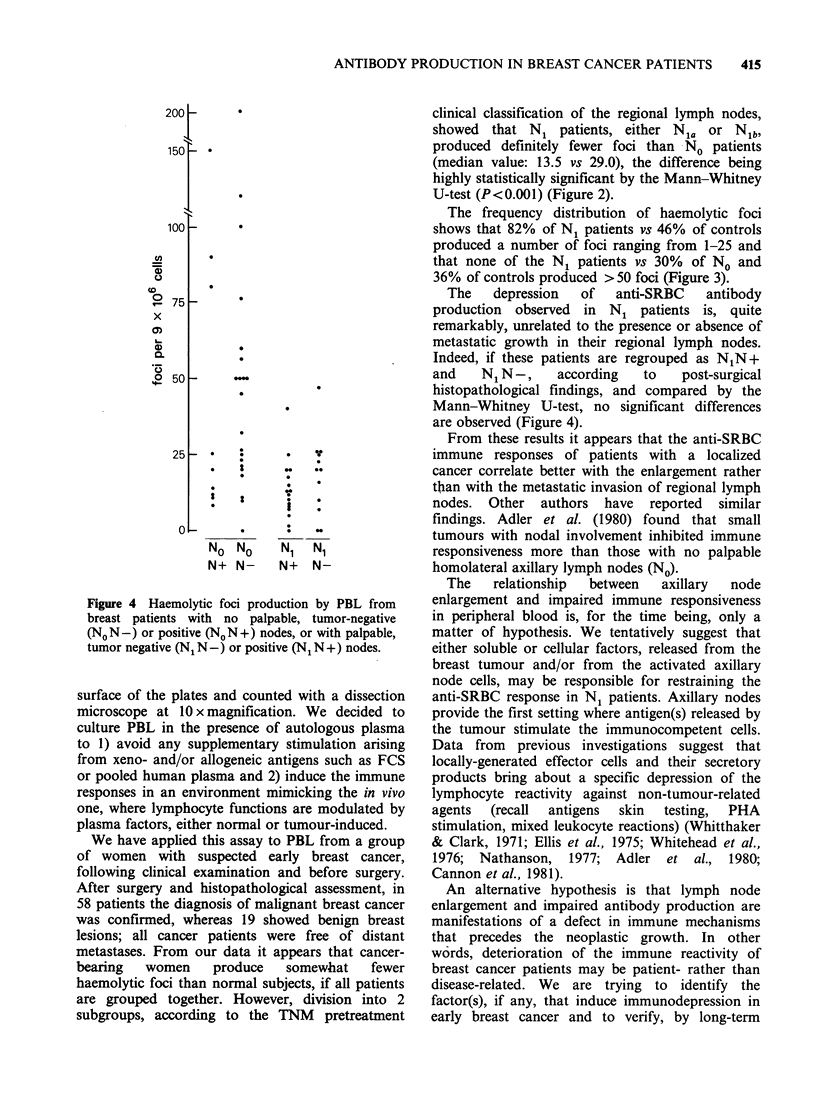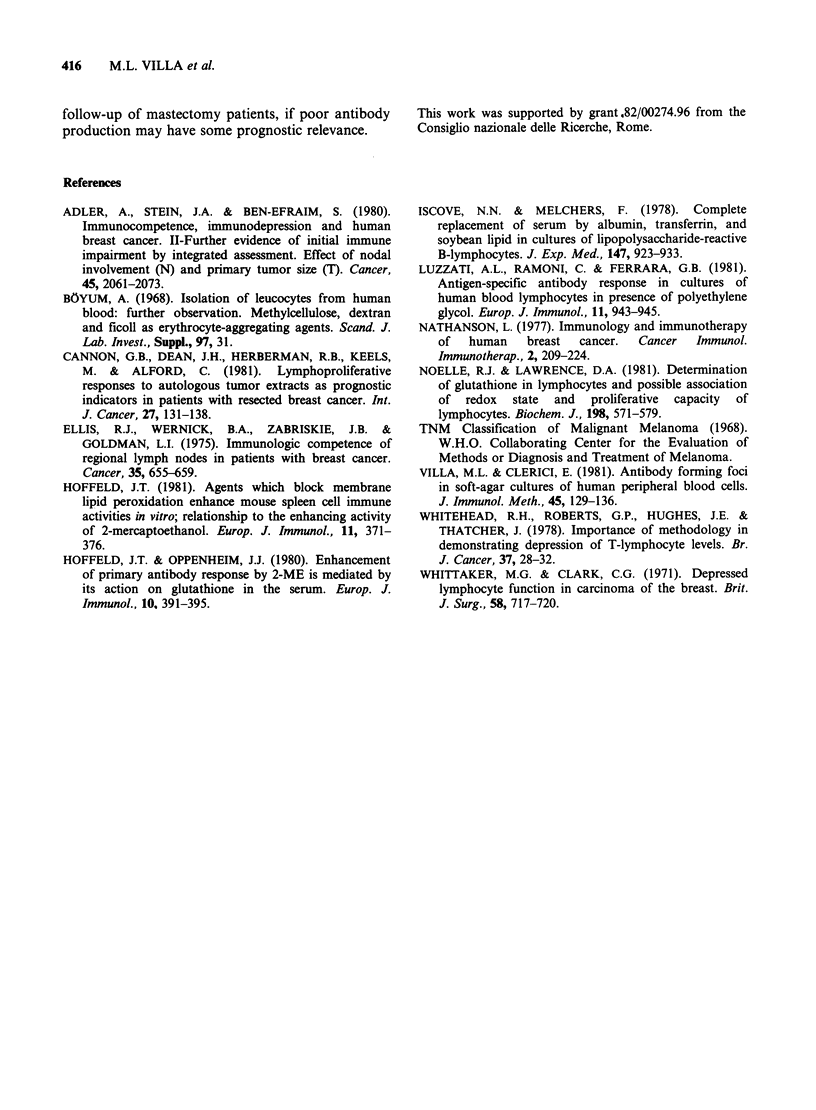# Antibody production in cultured blood lymphocytes from breast cancer patients.

**DOI:** 10.1038/bjc.1983.206

**Published:** 1983-09

**Authors:** M. L. Villa, M. Migliori, E. Clerici

## Abstract

Peripheral blood lymphocytes from female patients with early breast cancer were examined before surgery for their ability to develop a primary antibody response in vitro against sheep red blood cells in soft agar cultures containing autologous plasma. After 6 days incubation, foci of proliferating hemolysin-forming cells surrounded by a lytic area were detected on the surface of the plates and counted with a dissection microscope; this response was antigen-dependent and antigen-specific. We applied this assay to a group of women suffering from early breast cancer and devoid of distant metastases. From our data, it appears that if all the patients are grouped together, cancer-bearing women produce somewhat fewer (P less than 0.05) haemolytic foci than healthy controls. However, division of the cancer patients into two subgroups, according to the TNM pretreatment clinical classification of regional lymph nodes, generated an interesting finding: N1 patients (N1b or N1a) produced definitely fewer foci than N0 patients, and the difference was highly statistically significant (P less than 0.001). The depression of anti sheep red blood cell antibody production observed in N1 patients was unrelated to the presence or absence of metastatic growth in their regional lymph nodes.


*Br. J. Cancer* (1983), **48**, 411–416

### Antibody production in cultured blood lymphocytes from breast cancer patients

M.L. Villa, M. Migliori & E. Clerici

*Cattedra di Immunologia dell'Università di Milano and Istituto Nazionale per lo Studio e la Cura dei Tumori, via Venezian, 20133 Milano, Italy*

**Summary** Peripheral blood lymphocytes from female patients with early breast cancer were examined before surgery for their ability to develop a primary antibody response *in vitro* against sheep red blood cells in soft agar cultures containing autologous plasma. After 6 days incubation, foci of proliferating hemolysin-forming cells surrounded by a lytic area were detected on the surface of the plates and counted with a dissection microscope; this response was antigen-dependent and antigen-specific. We applied this assay to a group of women suffering from early breast cancer and devoid of distant metastases. From our data, it appears that if all the patients are grouped together, cancer-bearing women produce somewhat fewer ($P<0.05$) haemolytic foci than healthy controls. However, division of the cancer patients into two subgroups, according to the TNM pretreatment clinical classification of regional lymph nodes, generated an interesting finding: $N_1$ patients ($N_{1b}$ or $N_{1a}$) produced definitely fewer foci than $N_0$ patients, and the difference was highly statistically significant ($P<0.001$). The depression of anti sheep red blood cell antibody production observed in $N_1$ patients was unrelated to the presence or absence of metastatic growth in their regional lymph nodes.

A method for growing peripheral blood lymphocytes (PBL) in semisolid media and measuring specific antibody responses has been developed. PBL ($9\times10^6$) from normal donors, after 4–6 days of culture in soft-agar containing sheep red blood cells (SRBC) and autologous plasma, produce an average of 35–40 foci of anti-SRBC antibody-forming cells, which are easily scored because of surrounding areas of haemolysis (Villa & Clerici, 1981). This technique, which is relatively easy to perform and results of which are reproducible, has been devised to assay antibody production and immunoregulation in pathological conditions associated with immune disorders, particularly with malignant neoplasms.

The present paper deals with an attempt to further improve our culture method and to apply it to the study of the immune response in patients with breast carcinoma. Breast cancer has been chosen because it is linked with several distinctive alterations of immune responsiveness both in peripheral blood lymphocytes and regional lymph nodes, which take place from the early stages of disease (Nathanson, 1977). Our purpose was to correlate the primary anti-SRBC antibody response with the stage of the disease and the involvement of axillary lymph nodes.

Correspondence: M.L. Villa.

Received 3 May 1983; accepted 2 June 1983.

### Materials and methods

### *Patients*

The patient material of 77 cases was composed of females with suspected operable breast cancer, not previously treated elsewhere, observed, at the National Cancer Institute of Milan, from October 1980 to December 1981. Age distribution ranged between the 2nd and 6th decade, except for 2 patients aged 70 and 73 years. All patients were thoroughly examined by clinical and laboratory procedures, including complete skeletal X-rays survey, liver scan etc. Exact staging, based on the above evidence and on the postoperative histopathological report of the breast tumour and axillary lymph nodes, was determined and reported according to the rules of the "TNM classification of malignant tumours" (WHO, 1968). All blood samples were taken before biopsy and surgical operation, and investigators performing immunological assays were unaware of the pretreatment clinical classification of the case under examination.

### *Controls*

A control group of 60 healthy women, taken from blood donors, was evaluated in parallel for comparison. Patients and controls were rather evenly matched for age; 77% of controls and 71% of patients ranged between the 3rd and 5th decade.

© The Macmillan Press Ltd., 1983

*Lymphocyte separation*

Human PBL were isolated from heparinized blood by Ficoll Hypaque density centrifugation (Böyum, 1968). The plasma on the top of the gradient and the cells at the interface were harvested. The cells were washed twice in Dulbecco's modified Eagle Medium (DMEM) and suspended at a concentration of $3 \times 10^6 \, ml^{-1}$ in autologous plasma.

*Lymphocyte cultures*

The method of culturing PBL, previously described (Villa & Clerici, 1981), was used with some modifications. Briefly, double strength (2 ×) DMEM (Gibco) was supplemented with penicillin ($100 \, U \, ml^{-1}$), 3 mM reduced glutathione (GSH), 0.1% transferrin, and $15 \times 10^{-5}$ M 2-mercaptoethanol (2-ME). Cultures were set up in 60 mm plastic Petri dishes, and base layers were made by mixing equal volumes of 1% agar (Noble, Difco Lab. Inc., Detroit) and 2 × supplemented DMEM, to which autologous plasma (final concentration 10%) was added. Six ml aliquots of this mixture were poured into Petri dishes and allowed to gel at room temperature for 15 min. The overlay medium was prepared in such a way that each ml contained 0.33% agar in supplemented DMEM, $3 \times 10^6$ PBL in 15% autologous plasma, 10% washed SRBC, 8% polyethylene glycol (PEG) 6000 (Serva), and 0.3 μg% DEAE dextran (final concentrations in cultures are indicated). Three ml aliquots of this mixture, which was kept warm at 40°C, were then pipetted into the previously prepared base layers. After cooling at room temperature, the plates were incubated for 6 days at 37°C in a humidified incubator with constant flow of 10% $CO_2$ in air.

In the present paper, we have modified our previous culture technique (Villa & Clerici, 1981) to protect the responding lymphocytes from oxidative insults, arising from the environment (high $pO_2$) or from metabolism (production of superoxide anions and hydrogen peroxide by macrophages). We added transferrin, as suggested by Iscove and Melchers (1978), and reduced GSH, whose high content in foetal calf serum (FCS) accounts for its usefulness in murine cell cultures (Hoffeld & Oppenheim, 1980) and whose promoting activity on human PBL proliferation has been recently demonstrated (Hoffeld, 1981; Noelle & Lawrence, 1981). GSH, together with 2-ME, which prevents its spontaneous oxidation, allowed us to eliminate FCS and avoid the use of a special low oxygen-tension mixture in the incubation chamber.

*Haemolytic foci and cell-colony counting*

Haemolytic foci and cell-colony counting were done with a dissection microscope at × 10 magnification using an ocular micrometer. Counting was further facilitated by staining the entire plate with benzidine. For demonstration of cell colonies in the center of the plaques, cultures were stained with a diluted solution of acridine orange in PBS, pH 7.2 and observed under a fluorescence microscope.

### Results

*Clinical staging and post-surgical histopathological assessment*

In 58/77 patients, the diagnosis of breast carcinoma was confirmed histologically; 44 patients showed infiltrating ductal carcinoma, 10 infiltrating lobular carcinoma, 3 mixed ductal and lobular carcinoma, and 1 Paget carcinoma. In the remaining 19 patients, the lesions were found to be benign (fibrocystic diseases, epithelial duct hypertrophy and lipophagic granulomas).

Table I summarizes the clinical pretreatment classification of primary tumours (T), regional lymph nodes (N), distant metastases (M), and the post-surgical histopathological assessment of resected lymph nodes from patients with malignant disease. Most of the cases (88%) showed a primary mass that measured <5 cm ($T_0$, $T_1$ and $T_2$); about half of these patients had no palpable axillary lymph nodes ($N_0$), and the other half had palpable axillary lymph nodes ($N_1$), either suspected ($N_{1b}$) or not-suspected ($N_{1a}$) to be neoplastic. The post-surgical histological examination showed that 30% of $N_0$ patients and 57% of $N_1$ patients ($N_{1a}+N_{1b}$) had one or more tumour-positive nodes (N+). The remaining $N_1$ patients had hyperplastic nodes free of metastatic growth (N−). The enlarged lymph nodes showed sinus histiocytosis and/or hyperplasia of the paracortical areas.

*Anti-SRBC foci production*

Figure 1 shows the number of haemolytic anti-SRBC foci produced by women with breast carcinoma, benign breast lesions and normal controls. Cancer-bearing women produced less haemolytic foci than blood donors (median value, 20 *vs* 30), and the difference was statistically significant by the Mann–Whitney U-test ($P<0.05$). Although patients with benign breast lesions produced a relevant number of foci (median value, 40), they were excluded from statistical evaluation due to the limited extent of their group. No correlation between age and foci production was found in controls or in any of the patient groups.

**Table I** Clinical pretreatment classification of malignant breast cancers and postsurgical histopathological assessment of axillary lymph nodes.

| *Pretreatment clinical classification* | | *Postsurgical histopathology of resected axillary lymph nodes* | | | |
|---|---|---|---|---|---|
| | | *Tumour-negative nodes (N−)* | | *Tumour-positive nodes (N+)* | |
| *TNM stage groups* | *No. of cases* | *No. of cases* | *No. of nodes containing growth / No. of nodes examined* | *No. of cases* | *No. of nodes containing growth / No. of nodes examined* |
| $T_0N_0M_0$ | 14 | 11 | 0:16, 0:14, 0:11, 0:24, 0:18, 0:13, 0:10, 0:10, 0:17, 0:6, 0:10 | 3 | 3:18, 10:19, 1:14 |
| $T_1N_{1b}M_0$ | 5 | 3 | 0:22, 0:15, 0:18 | 2 | 7:18, 1:10 |
| $T_2N_0M_0$ | 11 | 7 | 0:25, 0:23, 0:20, 0:15, 0:25, 0:15, 0:14 | 4 | 2:15, 7:17, 1:15, 1:10 |
| $T_2N_{1a}M_0$ | 7 | 2 | 0:17, 0:12 | 5 | 1:17, 4:23, 1:17, 1:19, 3:20 |
| $T_2N_{1b}M_0$ | 14 | 7 | 0:19, 0:24, 0:15, 0:16, 0:12, 0:20, 0:22 | 7 | 1:24, 13:20, 3:15, 10:20, 7:20 18:33, 1:20 |
| $T_3N_0M_0$ | 2 | 1 | 0:9 | 1 | 2:20 |
| $T_3N_{1b}M_0$ | 1 | | | 1 | 11:18 |
| $T_4N_0M_0$ | 1 | 1 | 0:9 | | |
| $T_XN_0M_0$ | 1 | | | 1 | 8:15 |
| $T_XN_{1b}M_0$ | 1 | | | 1 | 1:13 |
| Paget $N_0$ | 1 | 1 | 0:21 | | |
| Total | 58 | 33 | | 25 | |

T: primary tumour extent; N: condition of regional lymph nodes; M: absence or presence of distant metastases (13). N+: patients with one or more nodes containing growth; N−: patients with all nodes free of metastases. The number of nodes containing growth, if any, and the total number of nodes examined are shown for each patient.

### *Relationship between lymph node assessment and anti-SRBC foci production*

Figure 2 summarizes the number of haemolytic foci produced by PBL of patients belonging to the $N_0$ and $N_1$ groups ($N_1 = N_{1a} + N_{1b}$), irrespective of the extent of their primary tumours. The data show a reduction in the antibody production by $N_1$ as compared to $N_0$ patients (median value, 13.5 *vs*. 29) and the difference was highly statistically significant ($P < 0.001$) by the Mann–Whitney U-test.

Figure 3 compares the frequency distribution of foci in the control group and in $N_0$ and $N_1$ patients: 46% of controls and 47% of $N_0$ patients *vs* 82% $N_1$ patients fell within the 1–25 range. The remaining $N_1$ patients (28%) were in the 26–50 range. None of the $N_1$ patients *vs* 36% of controls and 30% of $N_0$, respectively, produced >50 foci.

In Figure 4, the data from $N_0$ and $N_1$ groups are split according to the histopathology of axillary nodes into 4 subgroups, i.e., $N_1$N−, $N_1$N+, $N_0$N−, $N_0$N+. Statistical comparison between the subgroups $N_1$N− and $N_1$N+ showed that neither the presence nor the absence of metastases interfered with anti-SRBC foci production. As regards the $N_0$N− and $N_0$N+ subgroups, no statistical evaluation could be applied due to the unequal distribution of the data.

### Discussion

The present paper deals with the primary anti-SRBC antibody response in cultures of peripheral blood lymphocytes from patients with breast carcinoma. Human breast cancer offers a useful model because several women with early and advanced disease have been extensively studied in the past years by conventional *in vivo* and *in vitro* immunological assays (Nathanson, 1977). However, in the *in vitro* studies the lymphoid cells have been generally triggered by the use of polyclonal activators, rather than by defined antigens. This approach is fraught with the problem of extrapolating the results to antigen-specific immune mechanisms. We have overcome this obstacle and succeeded in developing a reproducible culture system for inducing antibody responses with human peripheral blood cells, based on the use of PEG 6000. This system is antigen-dependent and antigen-specific, as recently confirmed also by Luzzati *et al.* (1981), who were able to obtain an anti-SRBC response by adding PEG to their PBL cultures.

Briefly, PBL are cultured in soft agar together with SRBC and autologous fresh plasma in DMEM+8% PEG. After 6 days incubation, foci of proliferating haemolysin-forming cells surrounded by a lytic area are detected on the

**Figure 1** Haemolytic foci production by PBL from breast cancer patients, patients with breast lesions, and healthy controls.

**Figure 2** Haemolytic foci production by PBL from $N_0$ and $N_1$ patients. $N_0$, patients without palpable axillary nodes; $N_1$, patients with palpable axillary nodes.

**Figure 3** Frequency distribution (%) of anti-SRBC foci production by PBL from healthy controls (open bars), breast cancer patients with $N_0$ axillary nodes (cross-hatched bars), and with $N_1$ axillary nodes (solid bars).

**Figure 4** Haemolytic foci production by PBL from breast patients with no palpable, tumor-negative ($N_0N-$) or positive ($N_0N+$) nodes, or with palpable, tumor negative ($N_1N-$) or positive ($N_1N+$) nodes.

surface of the plates and counted with a dissection microscope at 10×magnification. We decided to culture PBL in the presence of autologous plasma to 1) avoid any supplementary stimulation arising from xeno- and/or allogeneic antigens such as FCS or pooled human plasma and 2) induce the immune responses in an environment mimicking the *in vivo* one, where lymphocyte functions are modulated by plasma factors, either normal or tumour-induced.

We have applied this assay to PBL from a group of women with suspected early breast cancer, following clinical examination and before surgery. After surgery and histopathological assessment, in 58 patients the diagnosis of malignant breast cancer was confirmed, whereas 19 showed benign breast lesions; all cancer patients were free of distant metastases. From our data it appears that cancer-bearing women produce somewhat fewer haemolytic foci than normal subjects, if all patients are grouped together. However, division into 2 subgroups, according to the TNM pretreatment clinical classification of the regional lymph nodes, showed that $N_1$ patients, either $N_{1a}$ or $N_{1b}$, produced definitely fewer foci than $N_0$ patients (median value: 13.5 *vs* 29.0), the difference being highly statistically significant by the Mann–Whitney U-test ($P<0.001$) (Figure 2).

The frequency distribution of haemolytic foci shows that 82% of $N_1$ patients *vs* 46% of controls produced a number of foci ranging from 1–25 and that none of the $N_1$ patients *vs* 30% of $N_0$ and 36% of controls produced >50 foci (Figure 3).

The depression of anti-SRBC antibody production observed in $N_1$ patients is, quite remarkably, unrelated to the presence or absence of metastatic growth in their regional lymph nodes. Indeed, if these patients are regrouped as $N_1N+$ and $N_1N-$, according to post-surgical histopathological findings, and compared by the Mann–Whitney U-test, no significant differences are observed (Figure 4).

From these results it appears that the anti-SRBC immune responses of patients with a localized cancer correlate better with the enlargement rather than with the metastatic invasion of regional lymph nodes. Other authors have reported similar findings. Adler *et al.* (1980) found that small tumours with nodal involvement inhibited immune responsiveness more than those with no palpable homolateral axillary lymph nodes ($N_0$).

The relationship between axillary node enlargement and impaired immune responsiveness in peripheral blood is, for the time being, only a matter of hypothesis. We tentatively suggest that either soluble or cellular factors, released from the breast tumour and/or from the activated axillary node cells, may be responsible for restraining the anti-SRBC response in $N_1$ patients. Axillary nodes provide the first setting where antigen(s) released by the tumour stimulate the immunocompetent cells. Data from previous investigations suggest that locally-generated effector cells and their secretory products bring about a specific depression of the lymphocyte reactivity against non-tumour-related agents (recall antigens skin testing, PHA stimulation, mixed leukocyte reactions) (Whitthaker & Clark, 1971; Ellis *et al.*, 1975; Whitehead *et al.*, 1976; Nathanson, 1977; Adler *et al.*, 1980; Cannon *et al.*, 1981).

An alternative hypothesis is that lymph node enlargement and impaired antibody production are manifestations of a defect in immune mechanisms that precedes the neoplastic growth. In other words, deterioration of the immune reactivity of breast cancer patients may be patient- rather than disease-related. We are trying to identify the factor(s), if any, that induce immunodepression in early breast cancer and to verify, by long-term follow-up of mastectomy patients, if poor antibody production may have some prognostic relevance.

This work was supported by grant ,82/00274.96 from the Consiglio nazionale delle Ricerche, Rome.

### References

ADLER, A., STEIN, J.A. & BEN-EFRAIM, S. (1980). Immunocompetence, immunodepression and human breast cancer. II-Further evidence of initial immune impairment by integrated assessment. Effect of nodal involvement (N) and primary tumor size (T). *Cancer*, **45**, 2061–2073.

BÖYUM, A. (1968). Isolation of leucocytes from human blood: further observation. Methylcellulose, dextran and ficoll as erythrocyte-aggregating agents. *Scand. J. Lab. Invest.*, **Suppl.**, **97**, 31.

CANNON, G.B., DEAN, J.H., HERBERMAN, R.B., KEELS, M. & ALFORD, C. (1981). Lymphoproliferative responses to autologous tumor extracts as prognostic indicators in patients with resected breast cancer. *Int. J. Cancer*, **27**, 131–138.

ELLIS, R.J., WERNICK, B.A., ZABRISKIE, J.B. & GOLDMAN, L.I. (1975). Immunologic competence of regional lymph nodes in patients with breast cancer. *Cancer*, **35**, 655–659.

HOFFELD, J.T. (1981). Agents which block membrane lipid peroxidation enhance mouse spleen cell immune activities *in vitro*; relationship to the enhancing activity of 2-mercaptoethanol. *Europ. J. Immunol.*, **11**, 371–376.

HOFFELD, J.T. & OPPENHEIM, J.J. (1980). Enhancement of primary antibody response by 2-ME is mediated by its action on glutathione in the serum. *Europ. J. Immunol.*, **10**, 391–395.

ISCOVE, N.N. & MELCHERS, F. (1978). Complete replacement of serum by albumin, transferrin, and soybean lipid in cultures of lipopolysaccharide-reactive B-lymphocytes. *J. Exp. Med.*, **147**, 923–933.

LUZZATI, A.L., RAMONI, C. & FERRARA, G.B. (1981). Antigen-specific antibody response in cultures of human blood lymphocytes in presence of polyethylene glycol. *Europ. J. Immunol.*, **11**, 943–945.

NATHANSON, L. (1977). Immunology and immunotherapy of human breast cancer. *Cancer Immunol. Immunotherap.*, **2**, 209–224.

NOELLE, R.J. & LAWRENCE, D.A. (1981). Determination of glutathione in lymphocytes and possible association of redox state and proliferative capacity of lymphocytes. *Biochem. J.*, **198**, 571–579.

TNM Classification of Malignant Melanoma (1968). W.H.O. Collaborating Center for the Evaluation of Methods or Diagnosis and Treatment of Melanoma.

VILLA, M.L. & CLERICI, E. (1981). Antibody forming foci in soft-agar cultures of human peripheral blood cells. *J. Immunol. Meth.*, **45**, 129–136.

WHITEHEAD, R.H., ROBERTS, G.P., HUGHES, J.E. & THATCHER, J. (1978). Importance of methodology in demonstrating depression of T-lymphocyte levels. *Br. J. Cancer*, **37**, 28–32.

WHITTAKER, M.G. & CLARK, C.G. (1971). Depressed lymphocyte function in carcinoma of the breast. *Brit. J. Surg.*, **58**, 717–720.